# Developing Laparoscopic Surgery on the Caribbean Island of St. Lucia: A Model for Public-Private Partnership

**DOI:** 10.7759/cureus.6011

**Published:** 2019-10-28

**Authors:** Shamir O Cawich, Daniel Kabiye

**Affiliations:** 1 Surgery, University of the West Indies, St. Augustine, TTO; 2 Surgery, Victoria Hospital, Castries, LCA

**Keywords:** laparoscopic, caribbean, saint lucia, surgery, mis

## Abstract

The first recorded laparoscopic operation in the Caribbean was a cholecystectomy performed in 1991. After a temporary peak in basic laparoscopic operations in subsequent years, the initial interest waned. While laparoscopic surgery was being popularized in the developed world, there was a stagnation in the Caribbean. There were many reasons for this stagnation, including a lack of surgical expertise, the negative attitudes of health-care workers, active opposition from surgical leaders, and equipment deficiencies, all exacerbated by the global financial recession in the early twenty-first century.

A similar situation existed on the Caribbean island of St. Lucia, where laparoscopic surgery remained relatively dormant. After a strong desire by community surgeons to incorporate advanced laparoscopy into surgical practice, surgical leaders in St. Lucia engineered a public-private partnership to achieve this. This review article evaluates the available data, documents the obstacles encountered, and explains the mechanisms to overcome these obstacles to incorporate advanced laparoscopy in St. Lucia. This information is important because it can serve as a template for other developing Caribbean countries.

## Introduction and background

The first recorded laparoscopic cholecystectomy (LC) was performed by Eric Mühe in Germany on September 12, 1985. In the subsequent decade, LC became accepted to the point where the National Institutes of Health issued this consensus statement: “Laparoscopic cholecystectomy provides a safe and effective treatment for most patients with symptomatic gallstones. Indeed, it appears to have become the treatment of choice.” This established LC as the gold standard operation for benign gallbladder disease.

The first recorded laparoscopic operation in the Caribbean was a cholecystectomy performed by Vijay Naraynsingh in Trinidad & Tobago in 1991 [1]. This was followed by reports of LC in Jamaica in 1993 [2] and simultaneously from Barbados [1] and the Cayman Islands in 1994 [3]. However, the initial interest in the Caribbean was not sustained. While laparoscopic surgery was being popularized in the developed world, there was a stagnation in the Caribbean [1]. As recently as in the year 2006, LC was performed uncommonly in Jamaica, at a rate of only two cases per month [2]. There were many reasons for the stagnation in the Caribbean, including a lack of surgical expertise [4], negative health care worker attitudes [5], active opposition from surgical leaders [6], and equipment deficiencies [7], and these were all exacerbated by the global financial recession in the early twenty-first century.

A similar situation existed on the Caribbean island of St. Lucia. Dr. Andrew Richardson performed the first laparoscopic operation in St. Lucia on October 2, 1998 - an elective cholecystectomy at a private facility, Tapion Hospital. But laparoscopy remained relatively dormant in St. Lucia over the subsequent decade. After a strong desire by community surgeons to incorporate advanced laparoscopy into surgical practice, surgical leaders in St. Lucia engineered a public-private partnership to achieve this. We document the obstacles encountered and the mechanisms to overcome these obstacles to incorporate advanced laparoscopy in St. Lucia. This information is important because it can serve as a template for other developing Caribbean countries.

Overview of health care in St. Lucia

**Figure 1:**
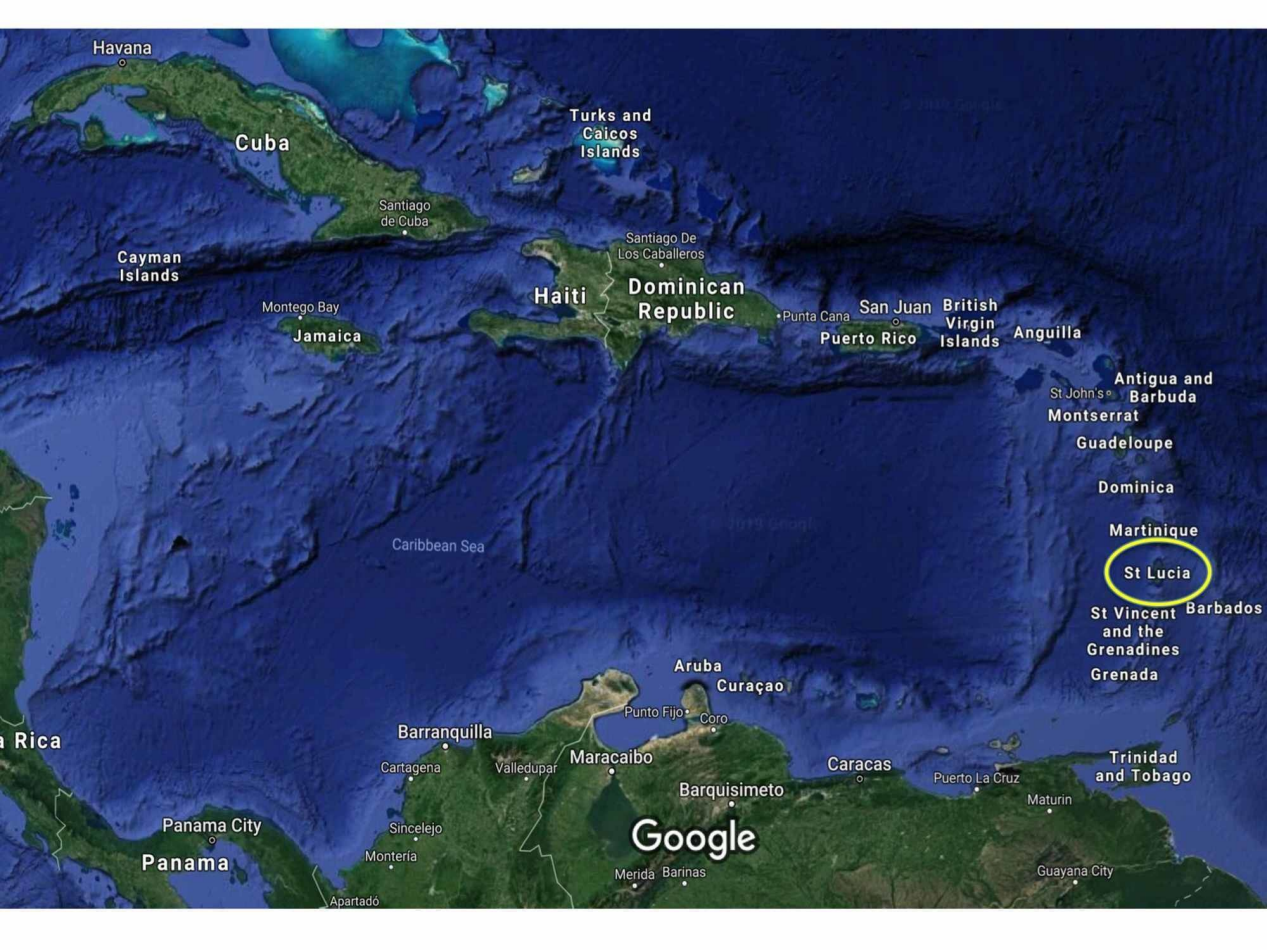
Satellite image of the Caribbean St. Lucia is an island nation in the Eastern Caribbean (encircled). Satellite image taken from Google images with permission.

The island of St. Lucia is a small independent nation in the Eastern Caribbean (Figure 1), with an estimated population of 178,000 persons at the last census [8]. It was a former colony of Great Britain that gained independence in 1979 [8]. It is now classified as a middle-income country with a gross national income per capita of $6,200.00 [9].

There are two parallel healthcare systems in St. Lucia: public healthcare systems that are government-funded and private health care systems that operate using a fee-for-service model. The Tapion Hospital is the only private facility that offers surgical care and is equipped with an operating room. At this facility, laparoscopic appendectomy, cholecystectomy, and tubal ligations are performed occasionally, but advanced cases are not attempted due to a paucity of trained personnel.

**Figure 2:**
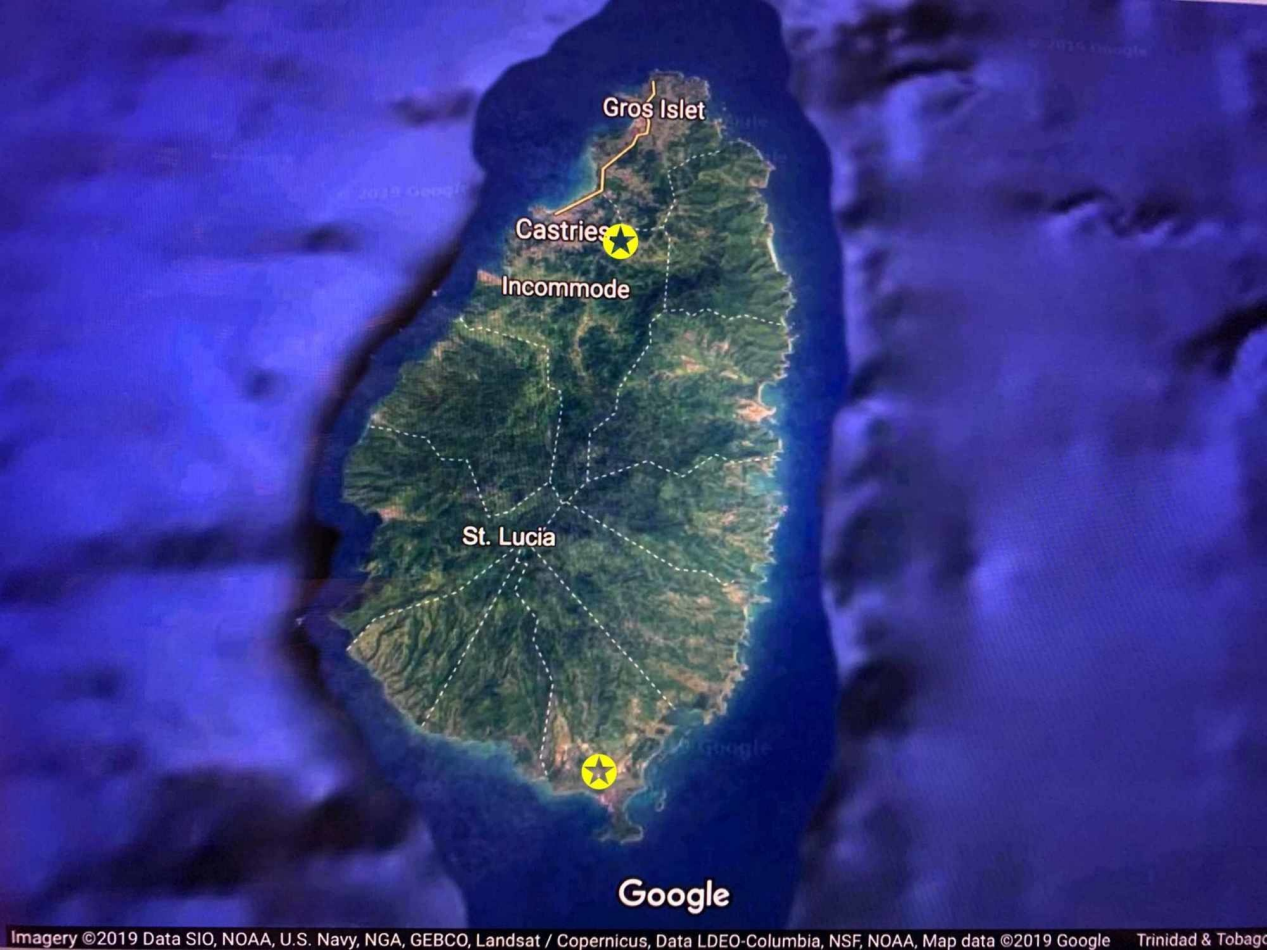
Satellite image identifying hospitals in St. Lucia Satellite image taken from Google satellite images showing the distribution of tertiary hospitals (star) across the island of St. Lucia.

The public healthcare system does not operate on a fee-for-service model. Instead, the Government of St. Lucia provides free healthcare to all legal residents on the island through a network of public health care facilities across the island. This network of healthcare facilities feeds patients to two tertiary referral hospitals across the island: The St. Jude Hospital located in the southern part of the Island and the Victoria Hospital in the northern part of the island (Figure 2).

Unlike the situation in developed countries, laparoscopic surgery has little purchase on the island of St. Lucia. After the fist LC was performed in 1998, many attempts were made to increase the popularity of laparoscopic surgery in St. Lucia. The first attempt was a laparoscopic workshop organized at a private facility, Tapion Hospital, in February 2009. In this workshop, a visiting gynecologist, Dr. John George of the Washington Hospital Centre, visited as a proctor to demonstrate laparoscopy and organized donated laparoscopic hardware and supplies. A few cases were done during the proctor’s tenure, but there was little continuity by local community surgeons. Further attempts were made in 2009 at the Tapion Hospital with a visiting proctor. Again, laparoscopic cases were performed in this workshop but there was little continuity of service when the proctors returned to their home hospitals.

A similar situation existed in the public healthcare system. The first laparoscopic operation was a cholecystectomy performed by Dr. Charles Greenidge in the year 2000. However, over the subsequent decade, basic laparoscopic operations were performed at very low volumes.

Surgical leaders at the Victoria Hospital, a public tertiary referral hospital, yearned to improve the standard of surgical care in the public healthcare system. But they recognized that there was still lethargy with respect to minimally invasive surgery (MIS), although multiple attempts had been made to promote laparoscopy. Surgical leaders at the Victoria Hospital decided to take a different approach to increase the popularity of laparoscopic surgery at their facility. We discuss the methods by which this was achieved and the challenges and successes of this exercise.

**Figure 3:**
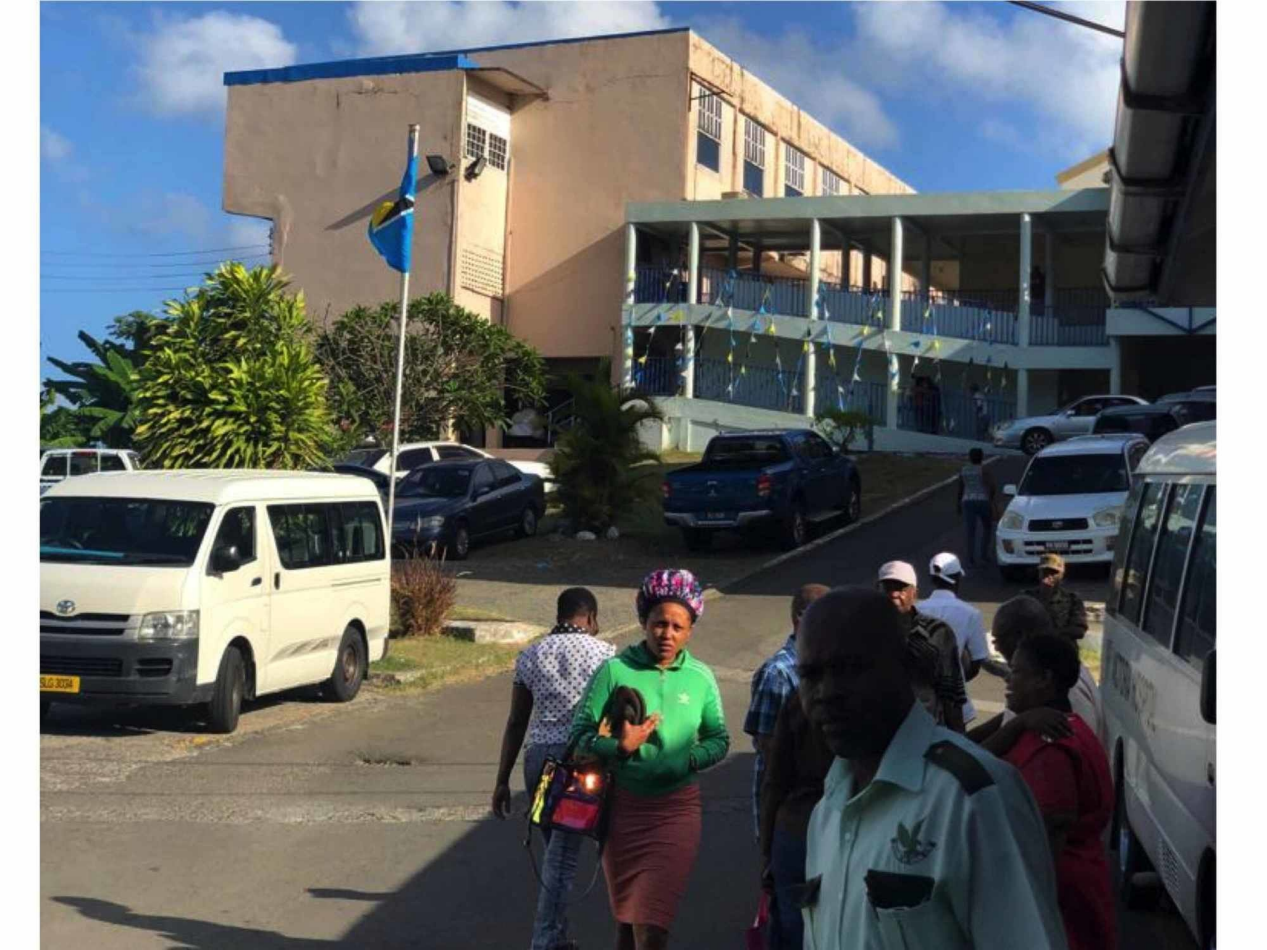
Victoria Hospital in St. Lucia A view of the entrance to Victoria Hospital - a 162-bed community hospital located in Castries, St. Lucia

**Figure 4:**
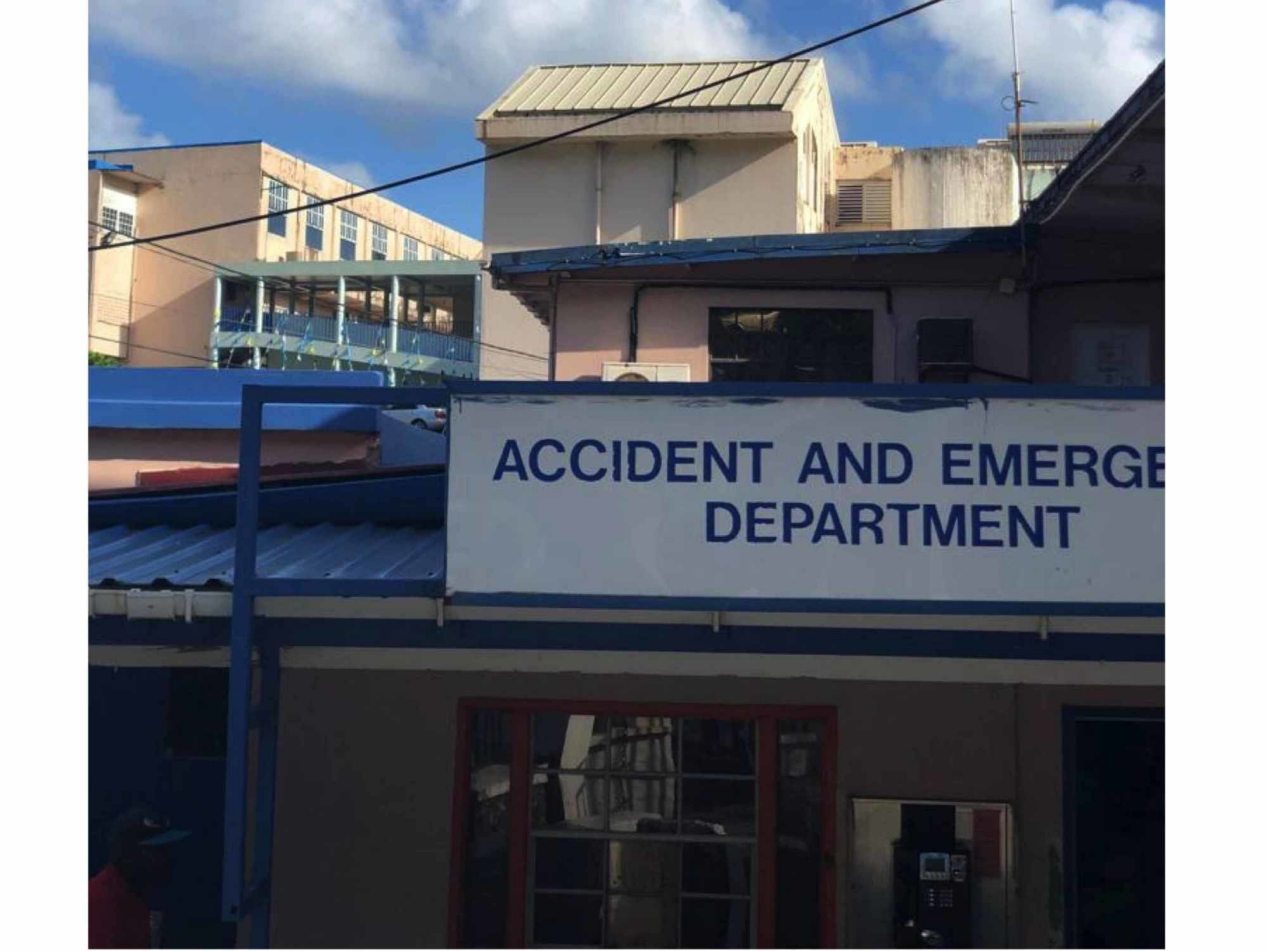
The Accident and Emergency department of Victoria Hospital in St. Lucia

The Victoria Hospital is a 162-bed community hospital located in Castries, St. Lucia (Figures 3-4). This facility was opened in 1887 and serves a catchment population of approximately 100,000 persons in northern St. Lucia. The general surgery department is staffed by three attending/consultant surgeons supported by 10 middle-grade house officers. The surgery department also includes four subspecialists, including urologists (1), otolaryngologists (1), and orthopedic surgeons (2). Together, the surgery department has access to 30 inpatient beds, three intensive care unit (ICU) beds, and two operating theaters and handles approximately 2,100 admissions on an annual basis.

## Review

Methods

(1) Formation of a Common Interest Group

Surgical leaders at Victoria Hospital opined that the country was not offering a standard of care in abdominal surgery. They noted that 20 years after laparoscopic surgery was first performed in St. Lucia, the volume of laparoscopic operations remained low. Therefore, a concerted effort was made to discuss this with general surgeons who unanimously voiced their interest in pursuing laparoscopy. These core individuals formed the Victoria Hospital Group (VHG).

The first step was for the VHG to meet, consolidate their vision, and develop an action plan. Hospital administrators and policymakers were kept involved at every stage. The VH medical director, Dr. Alisha Eugene, endorsed the movement. With endorsement by institutional administrators, it was time for the action plan to progress to phase two.

(2) Feasibility Study

The VHG understood the reality that advanced laparoscopy was simply not a priority for policymakers and that, in order to lobby effectively, they would need to have supportive data. Therefore, the VHG embarked on a one-year retrospective audit of the operating theater registers at the hospital between January 1, 2017, and December 30, 2017. The audit results are presented in Table 1.

**Table 1:** Abdominal operations performed at Victoria Hospital between January 1, 2017, and December 30, 2017 AP: anteroposterior

Procedure	Appendectomy	Cholecystectomy	Inguinal Hernia Repair	Ventral Hernia Repair	Hemicolectomy	Anterior Resection or AP Resections	Gastrectomy	Distal Pancreatectomy
Open Approach	30	12	32	13	3	2	4	0
Laparoscopic Approach	3	2	0	0	0	0	0	0
Total Number	33	14	32	13	0	0	0	0

There is a clear international consensus that the laparoscopic approach is superior to open surgery for gallstone disease [1-2], recurrent/bilateral/occult inguinal herniae [10], ventral herniorrhaphy [11], appendectomy [12], and colorectal resections [13]. There is also a great body of supportive evidence from the Caribbean region [14-17]. Despite this, laparoscopic resections only accounted for a small percentage of these operations in St. Lucia: appendectomy (9%), cholecystectomy (14%), inguinal herniorrhaphy (0), ventral herniorrhaphy (0%), and colectomies (0%).

The feasibility study provided local data that could now be used to support the call for improved standards of surgical care for users of this facility. Armed with this data, the VHG was now prepared to lobby healthcare policymakers, demonstrating that the population was not being offered the standard of care for abdominal surgery.

(3) Stakeholder Engagement

The VHG recognized that there is the potential for persons to erect barriers when they do not feel to be a part of a movement [18]. Therefore, the next step was to secure stakeholder buy-in to promote team spirit and one unified goal.

First, an informal survey was performed encompassing all tiers of healthcare workers who provide surgical care at the facility, including the nursing staff, surgical house officers, anesthetists, and clinical support staff. The healthcare workers were interviewed on their views on laparoscopic surgery at this facility. All interviewees were supportive of the initiative, but many expressed reservations because they were: not sufficiently exposed to laparoscopy (100%), unfamiliar with laparoscopic hardware and instrumentation (90%), unaware of the types of laparoscopic operations that could be performed (92%), perceived that setup was laborious (70%), perceived that there were high complication rates (65%), and they were not knowledgeable about the supportive data (80%). It was obvious that there needed to be an educational drive to curtail attitudes toward minimally invasive surgery. Therefore, the VHG recognized the need for and planned a lecture series to be held for healthcare workers in St. Lucia as part of their plan.

The VHG also attempted to engage health care workers by self-sponsoring four local healthcare providers to attend laparoscopic conferences in the region: the Curacao Laparoscopic Course in Curacao (one attendee), the Port of Spain General Hospital Laparoscopic Workshop in Trinidad & Tobago (one attendee), and the Caribbean Association of Endoscopic Surgeons (CaSES) workshop in Jamaica (two attendees). These educational events included didactic lectures in laparoscopic surgery and allowed the opportunity to “scrub in” for hands-on training in laparoscopic operations. There were good returns on these investments, as it generated interest and engaged the theater staff who became eager to start these procedures in their own facility.

(4) Financial Planning

It is accepted that a benefit of laparoscopic surgery is that it is cost-effective to the health care system in the long term although there are high start-up costs [15]. This, however, was a significant hindrance because the Government of St. Lucia was not in a position to meet the financial demands that this project required. Therefore, VHG found two ways to solicit sponsorship through private-public partnerships.

The VHG successfully lobbied the hospital administration citing current data from the facility. After letters were sent to the hospital advisory committee, they agreed to make a one-time grant available to assist in the purchase of a laparoscopic stack and reusable instrumentation using money from their annual budget. However, recognizing that the funds required supplementation, the administrators agreed to use the Benevolent Victoria Hospital Fund. This was a special-purpose account set up with the sole purpose of procuring laparoscopic hardware and consumables. It was serviced by monies collected from fees for elective operations, donations from private companies, as well as donations from the Government of Taiwan. Once there were sufficient funds, it was used to purchase laparoscopic hardware as determined by the medical advisory committee.

The VHG understood that multiple prior attempts were made to promote laparoscopy in St. Lucia, with limited success. Therefore, a part of their new plan was to organize a laparoscopic training workshop where proctors visited the Victoria Hospital to train local healthcare workers in their setting. This included plans for a full day of didactic lectures for all tiers of surgical support staff, followed by wet-lab training with laparoscopic simulators and then live operations where the surgical teams had the opportunity to “scrub in” while performing advanced operations. The audience would view, with two-way communication to the surgical proctor, during live operations. Obviously, this type of event would require significant funding that was not readily attainable through the healthcare budget or the depleted Benevolent Victoria Hospital Fund. Therefore, the VHG reached out to industry through their Caribbean subsidiaries.

A regional medical supply company met the challenge by committing full funding for a laparoscopic workshop in which they would provide laparoscopic towers, laparoscopic instrumentation, consumables, surgical educators, and a full team of biomedical engineers. The workshop was entirely funded by the private entity, amounting to an investment of well over USD 75,000.00 injected into the St. Lucia health care system.

(5) Organize Training Workshops

With support from the public-private partnership, the VHG group organized the inaugural “Victoria Hospital Group Laparoscopic Workshop” that took place on October 13-15, 2018. A complete medical education team led by Professor Shamir Cawich was flown into the island to execute the workshop.

**Figure 5:**
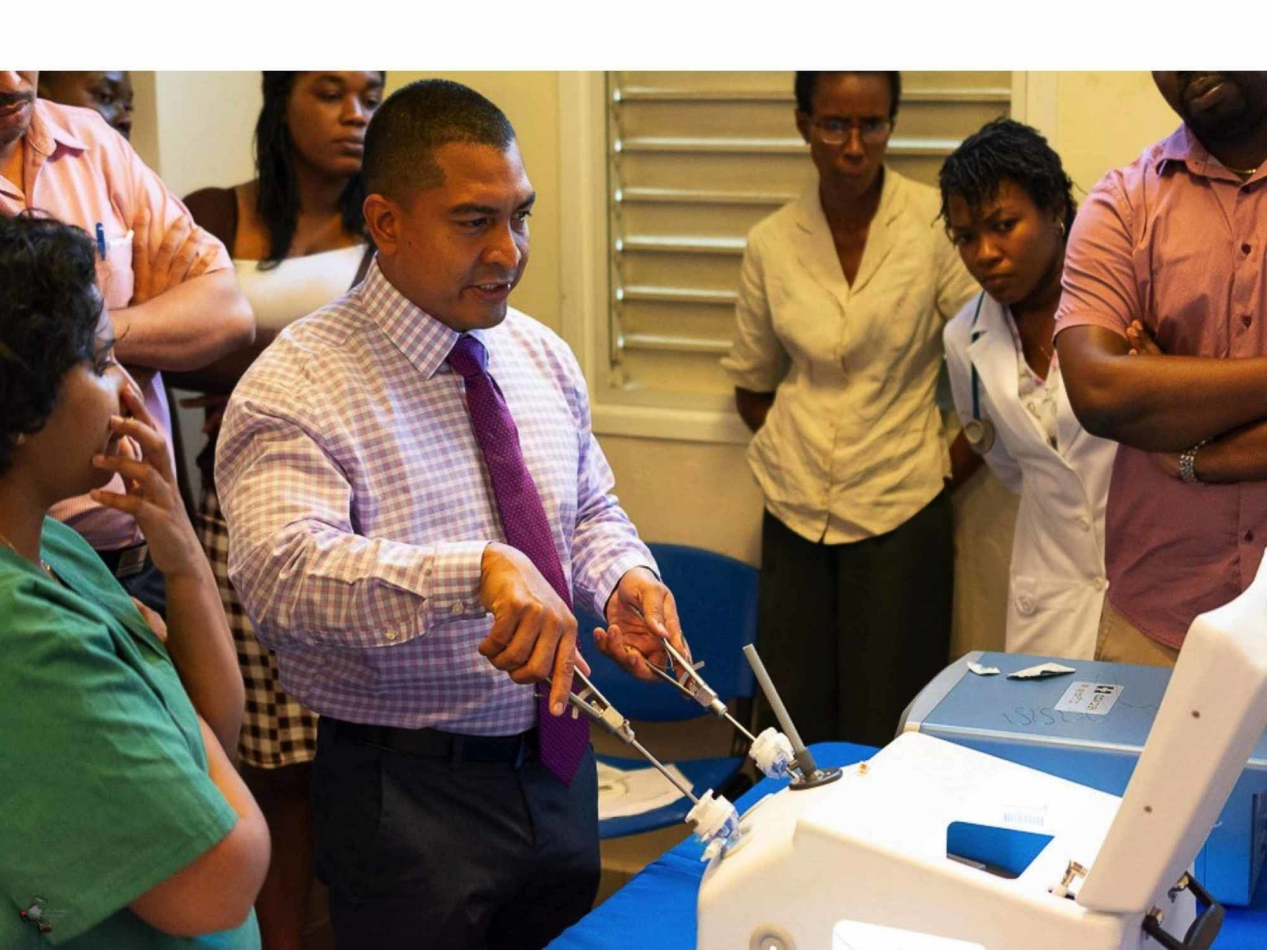
Skills lab teaching - laparoscopic suturing

The workshop delivered training to the entire healthcare team in their local environment in order to: (1) demonstrate that advanced laparoscopic operations are feasible in the local environment, despite existing challenges of the healthcare system, (2) train the entire healthcare team to set up and perform laparoscopic operations, and (3) generate local interest in laparoscopy. Therefore, there were lectures specifically geared for nurses, operating room technicians, attending grade surgeons, surgical staff, and anesthetic staff. In total, approximately 50 participants were trained in this activity. On the first day of the workshop, the Education team delivered didactic lectures on all aspects of laparoscopic surgery. The inclusion of a wide cadre of specialties on the medical education team was beneficial to engage the audience in all aspects of laparoscopy. There were also interactive training sessions using laparoscopic trainers where participants were proctored on a range of laparoscopic activities from peg transfers to suturing (Figure 5). The participants gained hands-on experience in using laparoscopic tools, including firing staplers in the wet lab, placing clips, and laparoscopic suturing. This was a valuable teaching tool to familiarize participants with laparoscopic instrumentation and techniques.

**Figure 6:**
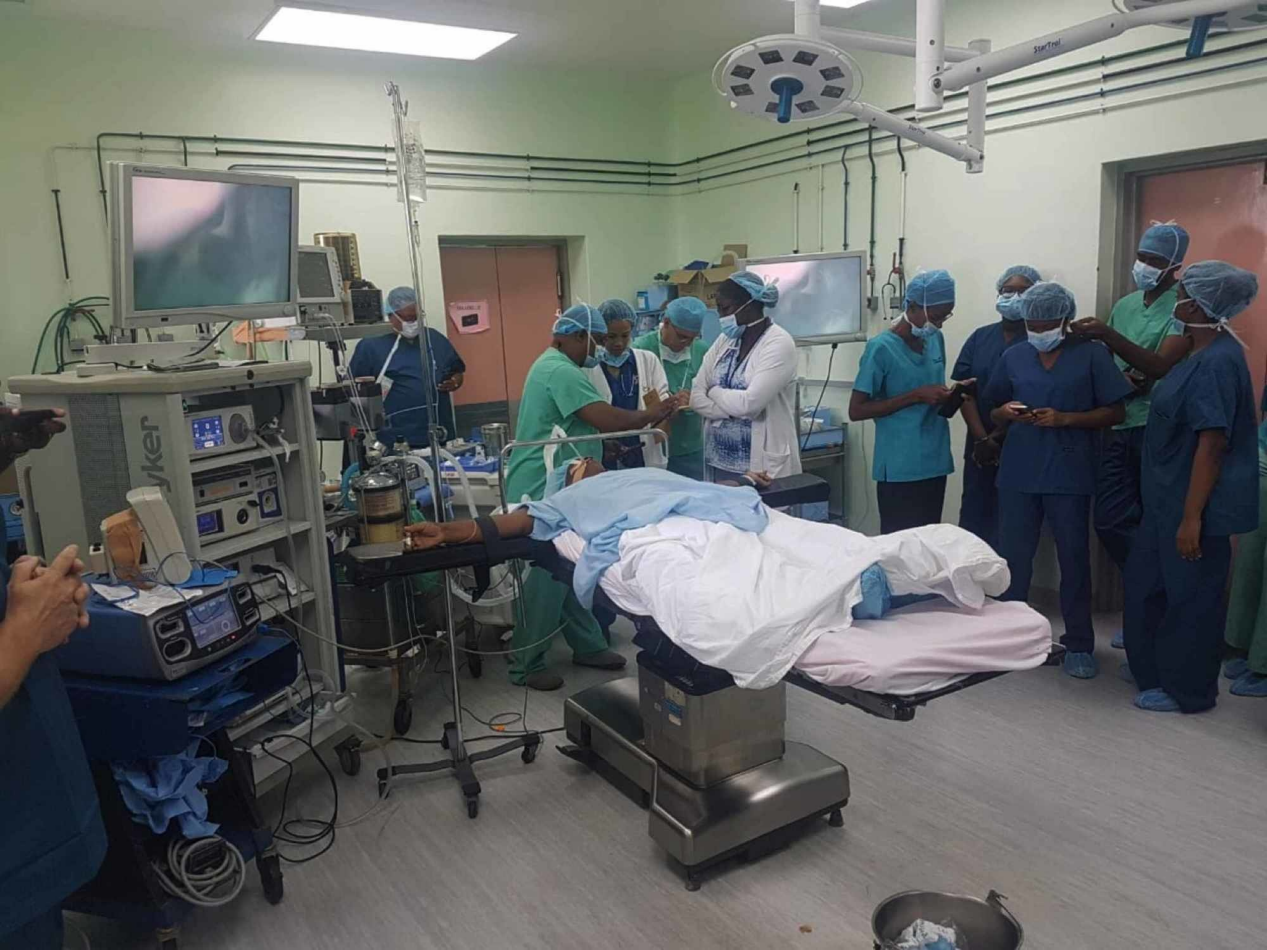
Live operation proctorship

The second day of the workshop comprised proctorship in the operating room for live surgery. In this segment of the workshop, the entire St. Lucian surgical team was proctored to perform laparoscopic operations, with the local team having the opportunity to scrub into and perform parts of the operations (Figures 6). Many operations were completed and some were performed for the first time in St. Lucia, including laparoscopic totally extraperitoneal (TEP) inguinal hernia repair, single incision laparoscopic surgery (SILS) cholecystectomy, and gastrectomy. This activity achieved all of the three intended goals, but the VHG realized that the effort could not stop at this point.

(6) Community Development

In addition to the engagement of health care workers and policymakers, the VHG realized that it was necessary to engage the community. Our anecdotal experience suggested that lobbying from laparoscopic surgeons alone would not be sufficient. It would require concurrent lobbying from the end-users of the health care system.

The local media supported the initiative by covering the workshop in newspapers, the electronic media, and televised interviews. We ensured that respected community surgeons from St. Lucia, and not the visiting team, were interviewed to educate the non-medical sector of the community on laparoscopic surgery [19] because we realized that they would be better able to garner support from local policymakers and the population they served. We hoped that this investment in community development would lead to a patient-driven demand for minimal access surgery.

We also appreciated the reality of healthcare politics: healthcare administrators or policymakers had the potential to destabilize the VHG momentum. Therefore, although their physical contributions were realistically minimal, we made sure that the media houses highlighted the contributions of the policymakers and interviewed and thanked them in their reporting. By highlighting the progress that was occurring in this small community hospital, we hoped that laparoscopic surgery would be elevated to the national stage, invigorating the laparoscopic revolution.

(7) Sustainability Planning

Approximately three months after the initial course, the staff at Victoria Hospital became familiar with the laparoscopic equipment and setup. Local surgeons gained experience with laparoscopic techniques and became confident with the new approach and technology. The entire VHG team had matured sufficiently together, and they were now able to perform these operations on their own without external support. In the five-month period after the workshop, a total of 21 laparoscopic operations were performed, including cholecystectomies (12), inguinal hernia repairs (2), appendectomies (2), bilateral tubal ligations (2), ovarian cystectomy (1), and diagnostic laparoscopies (2).

We recognize that VHG still has many hurdles to overcome and that there must be proper planning to ensure sustainability. At this point, a strong foundation has been laid that will allow this service to continue at Victoria Hospital: stakeholder engagement has positively impacted attitudes, community development and media involvement have created a patient-driven demand for these services, and investment in human resource has resulted in the development of local expertise in laparoscopy.

The public-private partnership provided support for the first workshop, but it was clear that the VHG group would need to find a mechanism for ongoing funding. Currently, the areas that we believe require attention are ongoing mentorship, reliable supply of consumables, and the continued audit of laparoscopic services.

In terms of continued education/mentorship, there are three bodies in the Caribbean that are geared toward continued education in laparoscopic surgery: the Caribbean College of Surgeons (CCOS), CaSES, and Curacao Foundation for Clinical Training. The local surgeons have all become active members of these associations and attend all regular training events. In addition, the concept of distance mentoring was developed in the Caribbean to serve as a continued educational and mentoring tool for new laparoscopic startup units [20] and this was one form of continued education at Victoria Hospital.

Although there are regional studies that have demonstrated the long-term cost-benefit advantage to laparoscopic surgery, there is a need to collect local data from St. Lucia. Although the VHG has already made the case to the local administration that there are cost benefits in the long term, regular reminders by presenting audit data will reinforce this fact and ensure the continued engagement of the policymakers. In addition, continued dialogue and data sharing with private entities who supported the movement would also ensure continued dialogue to show that there was some worth from the investment. Additionally, the local group must recognize the invaluable input from the industry and continue to engage them to allow them to recognize their long-term investment.

Regarding the uninterrupted supply of consumables, we assumed that the health care system may not have the financial means to continually procure consumables. Therefore, a proposal was made to re-open the Benevolent Victoria Hospital Fund where 20% of income from laparoscopic surgery would be deposited to this fund with the explicit purpose of refreshing consumables for the VHG. This is under consideration by the hospital administration.

Some potential ideas for sustainability included a proposal for regional medical suppliers to provide consumables on consignment or having patients purchase the necessary consumables for their operations, which would be supplemented by the government-funded services.

In addition, a business plan and project proposal were created and sent to corporate entities, service clubs, and non-governmental organizations seeking extra-budgetary funding. We also use reusable instrumentation and minimize the cost of disposables by relying more on intra-corporeal suturing and tying instead of costly disposables. Finally, proctors from the region have been engaged to teach low-cost techniques that were sustainable in a developing nation setting. These business plans have been presented to the hospital administrators and are being considered to provide continued finance for the VHG laparoscopic service.

## Conclusions

Despite many challenges, a laparoscopic surgery service has finally been started at this small community hospital in St. Lucia. There were several obstacles along the way: inadequate funding, absence of laparoscopic hardware, and suboptimal health care worker attitudes. But, driven by a desire to improve the standard of surgical care provided to their patients, the VHG group surmounted these obstacles and successfully implemented a laparoscopic service. The lessons learned are useful for other developing nations where laparoscopy remains in its infancy. The tasks that must not be ignored are stakeholder engagement, staff training, effective media involvement, community development, proper financial planning, capitalizing on public-private partnerships, and sustainability planning.
